# The Vertical and Horizontal Pathways in the Monkey Retina Are Modulated by Typical and Atypical Cannabinoid Receptors

**DOI:** 10.3390/cells10113160

**Published:** 2021-11-13

**Authors:** Joseph Bouskila, Maxime Bleau, Catarina Micaelo-Fernandes, Jean-François Bouchard, Maurice Ptito

**Affiliations:** 1 School of Optometry, University of Montreal, Montreal, QC H3Y 1P1, Canada; joseph.bouskila@umontreal.ca (J.B.); maxime.bleau.1@umontreal.ca (M.B.); catarina.fernandes@umontreal.ca (C.M.-F.); jean-francois.bouchard@umontreal.ca (J.-F.B.); 2 Behavioral Science Foundations, Eastern Caribbean, Estridge KN0101, Saint Kitts and Nevis; 3 Department of Neuroscience, Copenhagen University, 2200 Copenhagen, Denmark; 4 Department of Neurology and Neurosurgery, Montreal Neurological Institute, McGill University, Montreal, QC H3A 2B4, Canada

**Keywords:** retina, typical cannabinoid receptors, atypical cannabinoid receptors, immunohistochemistry, electroretinography, monkeys, visual system

## Abstract

The endocannabinoid (eCB) system has been found in all visual parts of the central ner-vous system and plays a role in the processing of visual information in many species, including monkeys and humans. Using anatomical methods, cannabinoid receptors are present in the monkey retina, particularly in the vertical glutamatergic pathway, and also in the horizontal GABAergic pathway. Modulating the eCB system regulates normal retinal function as demonstrated by electrophysiological recordings. The characterization of the expression patterns of all types of cannabinoid receptors in the retina is progressing, and further research is needed to elucidate their exact role in processing visual information. Typical cannabinoid receptors include G-protein coupled receptor CB1R and CB2R, and atypical cannabinoid receptors include the G-protein coupled receptor 55 (GPR55) and the ion channel transient receptor potential vanilloid 1 (TRPV1). This review focuses on the expression and localization studies carried out in monkeys, but some data on other animal species and humans will also be reported. Furthermore, the role of the endogenous cannabinoid receptors in retinal function will also be presented using intraocular injections of known modulators (agonists and antagonists) on electroretinographic patterns in monkeys. The effects of the natural bioactive lipid lysophosphatidylglucoside and synthetic FAAH inhibitor URB597 on retinal function, will also be described. Finally, the potential of typical and atypical cannabinoid receptor acti-vity regulation in retinal diseases, such as age-related macular degeneration, diabetic retinopathy, glaucoma, and retinitis pigmentosa will be briefly explored.

## 1. Introduction

**Figure 1:**
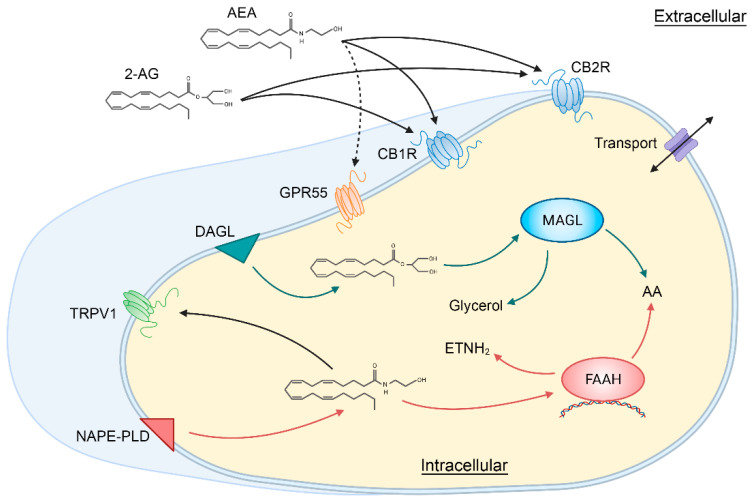
Schematic illustration showing the main components of the classical endocannabinoid system. 2-AG, 2-arachidonylglycerol; AA, arachidonic acid; AEA, anandamide or N-arachidonoylethanolamine; CB1R, cannabinoid receptor type 1; CB2R, cannabinoid receptor type 2; DAGL, Diacylglycerol lipase; ETNH_2_, ethanolamine; FAAH, fatty acid amide hydrolase; GPR55, G protein-coupled receptor 55; MAGL, monoacylglycerol lipase; NAPE-PLD, N-acyl phosphatidylethanolamine phospholipase D; TRPV1, transient receptor potential vanilloid type 1. Created with BioRender.com and based on [1].

The organization of the normal retinal mosaic is presented in the introductory chapter (Ptito et al., 2021, this volume) and we will concentrate here on the general components of the endocannabinoid (eCB) system. The eCB system is comprised of various components. There are 2 main types of receptors, cannabinoid receptors type 1 (CB1R) and type 2 (CB2R); endogenous ligands acting upon cannabinoid receptors, termed endocannabinoids (eCBs), mainly anandamide (AEA) and 2-arachidonylglycerol (2-AG); and enzymes metabolizing eCBs, particularly N-acyl phosphatidylethanolamine phospholipase D (NAPE-PLD) and diacylglycerol lipase (DAGL) for the synthesis of eCBs and fatty acid amide hydrolase (FAAH) and monoacylglycerol lipase (MAGL) for the degradation of eCBs [1] (Figure 1).

ECBs are derived from long-chain polyunsaturated fatty acids (amides, esters and ethers), mainly arachidonic acid, found in the central and peripheral nervous systems [2,3]. While FAAH converts AEA to ethanolamine and arachidonic acid, MAGL converts 2-AG to glycerol and arachidonic acid. AEA and 2-AG are the most studied eCBs, but others are still being unveiled [4]. Since THC, the main psychoactive compound of cannabis, binds to the same CB1R and CB2R as eCBs, the latter mimic most of the central and peripheral effects of cannabis [5,6].

**Figure 2:**
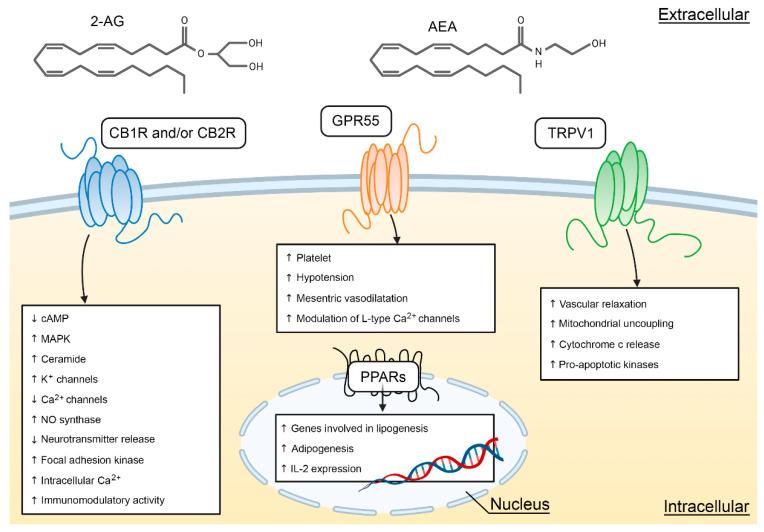
Cellular targets and signaling pathways of AEA and 2-AG. This figure illustrates the main cellular effects for CB1R/CB2R, GPR55, and TRPV1. Redrawn from [19] and created with BioRender.com.

CB1R and CB2R are the two classical cannabinoid receptors with seven transmembrane passages and coupled to the G proteins. They are most often coupled to the G_i_/G_o_ proteins, inhibit adenylate cyclase, and regulate the calcium and potassium-type ion channels [7] (Figure 2). In 1990, CB1R was identified and cloned [8]. In 1993, CB2R was discovered [9] and, two years later, cloned [10]. Given their differential expression within the body, they have usually been studied independently. CB1R is abundantly expressed in the CNS of all vertebrates [11] and have been ascribed multiple roles, particularly, in the development of the brain (e.g., axon growth) [12,13,14,15] and visual functions [16,17,18].

Recently, several studies showed that there may be other cannabinoid receptors, such as the G protein-coupled receptor 55 (GPR55), the ionotropic transient receptor potential vanilloid 1 (TRPV1) and the nuclear peroxisome proliferator-activated receptor (PPAR) (see chapter 3 of [19]). Accordingly, cannabinoid receptors have been divided into typical ad atypical receptors. The typical cannabinoid receptors are CB1R and CB2R, and non-CB1R/CB2R cannabinoid effects define the atypical cannabinoid receptors, mainly GPR55 and TRPV1. Figure 2 shows the main signaling channels for CB1R, CB2R, GPR55, TRPV1, but also PPAR, a receptor that has not been extensively studied in the retina. Cannabinoids, including phytocannabinoids, endocannabinoids, and exogenous synthetic cannabinoid modulators, bind to the traditional two types of cannabinoid receptors CB1 and CB2, but also, to other GPCR and ion channels. In the past decade, it has been reported that both, typical and atypical cannabinoid receptors, are expressed in the visual system of rodents, monkeys, and humans [18,20,21].

## 2. The Retinal Endocannabinoid System

**Figure 3:**
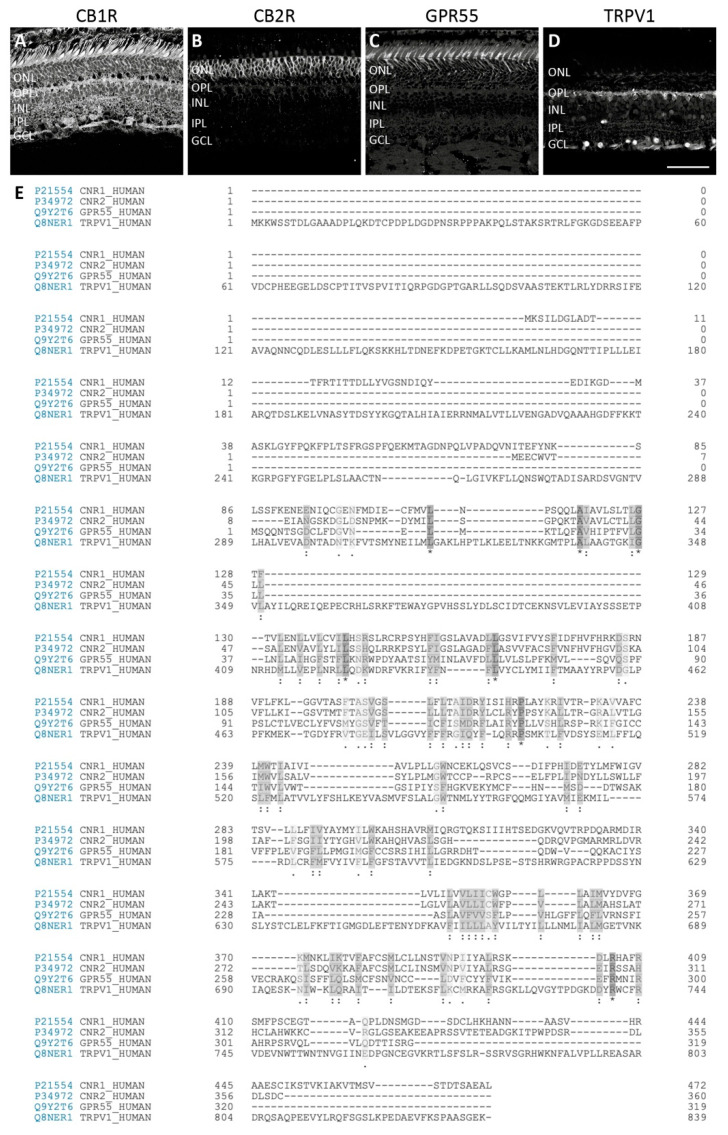
Comparison of typical and atypical cannabinoid receptors. Confocal immunofluorescence images of vervet monkey retinal sections (**A**–**D**) and human protein sequence alignment (**E**) of CB1R, CB2R, GPR55, and TRPV1. CB1R is localized in neural components, with very weak (albeit absence) in rods [16] (**A**). CB2R is strictly expressed in the glial components, the Müller cells [29] (**B**). GPR55 is found exclusively in rods, with the most prominent staining in the inner segments [28] (**C**). TRPV1 is expressed in the lateral pathway, particularly horizontal cells and amacrine cells [27] (**D**). Protein alignment show conserved regions that may be important for the structural and functional effects of cannabinoids. Scale bar = 75 µm.

Expression studies have shown that CB1R, CB2R, GPR55, and TRPV1 are present in the retina of multiple species [13,14,17,18,21,22,23,24,25,26], but their differential expression has been found solely in the monkey retina [16,27,28,29,30]. More precisely, our studies demonstrate that these receptors are distributed in the vertical and horizontal pathways of the vervet monkey retina. It is important to stress out that good, specific, validated antibodies are important for proper characterization of cannabinoid expression patterns, e.g., targeting CB2R using immunohistochemistry is controversial in the literature [31,32]. Similarly, commercially available TRPV1 antibodies generate non-specific labeling in the retina of some species [33]. Using the non-human primate model and antibodies directed against human epitopes for cannabinoid receptors, in our experience, was found adequate [30,34]. In line with this, we compared the alignment of the human sequence for these 4 receptors (Figure 3).

### 2.1. Typical Cannabinoid Receptors CB1R and CB2R

#### 2.1.1. Anatomical Localization and Function

**Figure 4:**
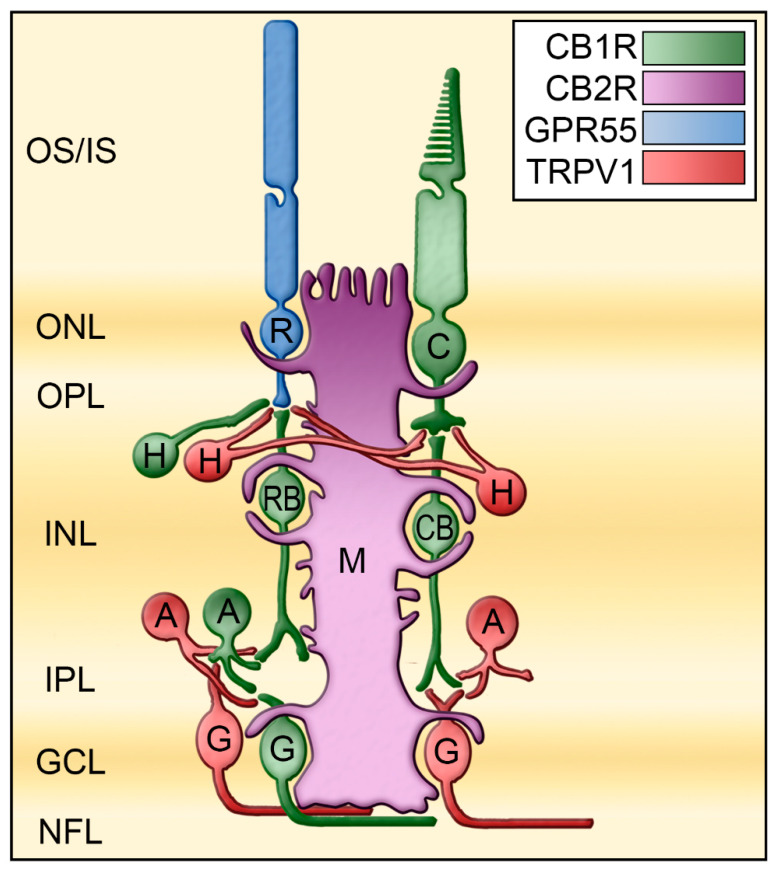
Mapping of the receptors CB1R, CB2R, GPR55, and TRPV1 in the monkey retina. These receptors are differently expressed in the retina of monkeys. These results are compiled from several published articles [16,27,28,29]. CB1R is represented in green, CB2R in magenta, GPR55 in blue, and TRPV1 in red. A, amacrine cells; C, cone photoreceptors; CB, cone bipolar cells; G, ganglion cells; GCL, ganglion cell layer; H, horizontal cells; INL, inner nuclear layer; IPL, inner plexiform layer; IS, photoreceptors inner segments; M, Müller cells; NFL, nerve fiber layer; ONL, outer nuclear layer; ONL, outer plexiform layer; OS, photoreceptors outer segments; R, rod photoreceptors; RB, rod bipolar cells. Redrawn from [27].

Traditionally, CB1R has been associated with the CNS and CB2R with the immune system. This idea was then developed into CB1R in neurons to modulate neurotransmitter release, and CB2R in glial cells to modulate cytokine release. While this differential expression is not present in the rodent retina, it appears to be present in the vervet monkey retina. In healthy monkeys, the presence of CB1R in the neuroretina has been well characterized [16]. CB1R is highly expressed in the foveal region, owing to its abundant expression in cone photoreceptors. It is also present throughout the vertical pathway consisting of rod and cone photoreceptors, bipolar cells, and ganglion cells, and at a lesser degree in the horizontal pathway consisting of horizontal cells and amacrine cells. CB2R is exclusively expressed in the major glial element of the retina (Müller cells), extending from the outer limiting membrane to the inner limiting membrane [29]. Furthermore, localization of their associated metabolic enzymes suggests that eCBs are synthesized and released in the synapse surrounding the neurons from which they are released [13,18]. This dual CB1R/CB2R expression pattern already suggests differential retinal functions (Figure 4).

**Figure 5:**
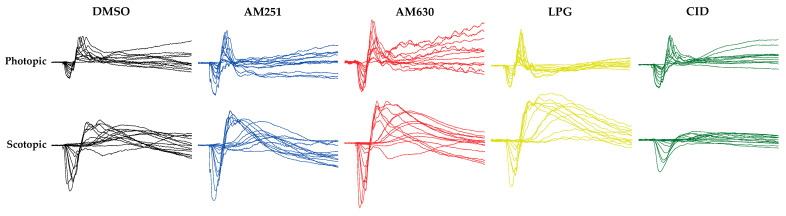
Raw photopic and scotopic ERGs in the different drug injection groups. The effect of modulating CB1R, CB2R, or GPR55 in the monkey retina. The intravitreal injection of AM251 (blue), an inverse agonist of CB1R, or AM630 (red), an inverse agonist of CB2R, causes an increase of the ERG photopic (under a 30 cd/m^2^ white background-adapting field) and scotopic (recorded under dark-adapted conditions in a light-tight room) responses, compared to the vehicle, dimethyl sulfoxide (DMSO, in black) [20]. The intravitreal injection of lysophosphatidylglucoside (LPG), an agonist of GPR55 (yellow), causes an increase of the scotopic response, but not of the photopic response. Conversely, the intravitreal injection of CID16020046 (CID), an antagonist of GPR55, causes a decrease of the scotopic response, but not of the photopic response (green).

The hypothetical function of cannabinoid receptors in the monkey retina could be extended to the human retina given the strong similarities in its anatomical organization. Intraocular injections of specific blockers for CB1R and CB2R in the monkey retina lead to an increase of photopic and scotopic responses (Figure 5; [20]).

#### 2.1.2. Interspecies CB1R/CB2R Comparison

Several pieces of evidence lead us to believe that these receptors are not expressed in the retina in a similar way across species, e.g. the protein sequence of mouse CB2R is different from that of the primate. There are some major differences in protein sequences as one goes up the animal ladder. CB1R and CB2R are unique to Chordata (phylum of animals that has a notochord, a cartilaginous lamella of mesodermal origin located on the dorsal side of the animal). Furthermore, synthetic and degradative enzymes are present in several species of the animal kingdom [35]. It is possible that receptor-acting proteins appeared much later than eCBs. Although the expression patterns of some eCBs components like CB1R and FAAH are similar in different species, this is not the case for CB2R and NAPE-PLD [34]. As the expression of CB2R in the mouse retina [22] is different from the monkey [29], we studied several components of the eCB system known to date in the mouse retina, monkeys and treeshrews (an intermediate species in the phylogenetic tree). In addition, the expression of this system has been compared in the retina of two types of monkeys, namely vervets and macaques. In the retina of mice, treeshrews, macaques, and vervets, CB1R, and its associated eCB degrading enzyme FAAH, have an overlapping expression pattern [34]. This suggests that degradation of eCBs may occur in the same CB1R positive retinal cell. For CB2R, some important expression differences are present. In the mouse rod-dominated retina, CB2R is expressed in the neuro-retina, in photoreceptors, horizontal cells, bipolar cells, amacrine cells, and ganglion cells, but not in Müller cells [22]. In the cone-dominated retina of treeshrews, CB2R is expressed in neuroretina and also in Müller cells. In the duplex retina of vervet and macaque monkeys, CB2R is found exclusively in Müller cells. One possible explanation for this transition of CB2R distribution (diffuse neuronal expression vs. specific expression in the retinal glia) is that this receptor has assumed a strategic place to exert the role of potassium modulator during the evolution of the retina. Interestingly, the synthetic enzyme NAPE-PLD also has an expression pattern specific to each of these species. In mice, NAPE-PLD is expressed in the neuroretina [34]. In the Tree shrew, NAPE-PLD is expressed strongly in the external retina and weakly in the internal retina [34]. In monkeys, NAPE-PLD is expressed only at the level of photoreceptors, cones and rods [34]. The bioactive lipid molecules, N-acyl ethanolamines (NAE), are synthesized by NAPE-PLD from cell membrane phospholipids. This interspecies retinal expression difference is noted despite the conserved protein sequence of NAPE-PLD, unlike CB2R. This variation may be of importance to NAPE-PLD constructs other than eCBs. This enzyme synthesizes several molecules such as anandamide (an eCB), but also N-palmitoylethanolamine (an anti-inflammatory agent; [36]) and Noleoylethanolamine (an anorexic agent; [37]). NAPE-PLD can even have proapoptotic effects [38]. In addition, the products of this enzyme, NAE, are present in axons and regulate postsynaptic neuronal activity by acting as anterograde synaptic signaling molecules ([39]. Finally, the expression of NAPE-PLD in monkey photoreceptors suggests a direct role of NAEs in primate phototransduction. But this remains to be verified experimentally. The expression patterns of DAGL and MAGL in the retina of mice, treeshrews and monkeys are similar, but have different signal intensities [34,40]. As eCBs are synthesized and degraded around their receptors, expression of DAGL and MAGL must be found in the vicinity of CB2R. In the mouse retina, these two enzymes in addition to CB1R and CB2R are expressed in an overlapping manner and therefore may exert a self-regulatory role in each of the cells of the retina. In the retina of tree shrews and monkeys, the expression of enzymes of the eCB system is found in a complementary manner. The eCB system may have specialized in parallel with the increasing complexity of the visual system to adopt a strategic position for the modulation of visual activity

**Figure 6:**
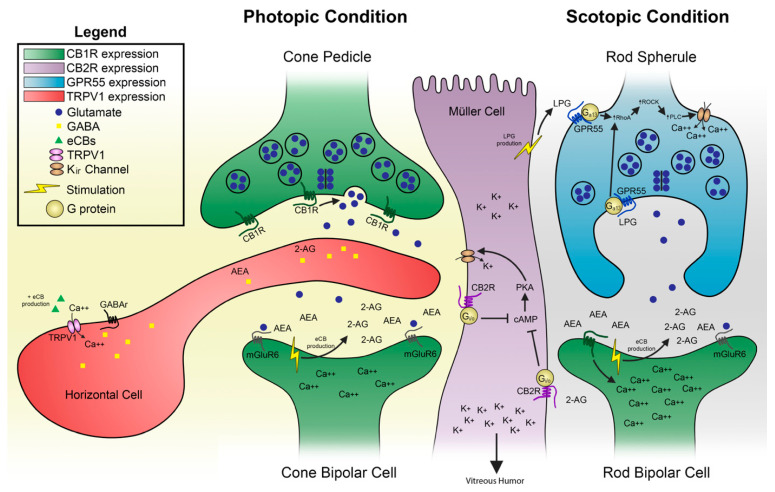
The hypothetical molecular mechanisms for typical and atypical cannabinoid receptors action in the retina is illustrated at the photoreceptor-bipolar cell synapse. This may also exist in several other synapses in the visual where this differential expression pattern exists. LPG, as suggested in previous models [30,41], could be produced by Müller cells. Adapted from [30].

The molecular basis of CB1R/CB2R action in retinal function can be explained by a model recently published [30]. In photopic conditions, when cones are stimulated by light, the ionic channels are inhibited, a process known as the “inhibition of the retinal dark currents.” The resulting phototransduction reduces the glutamate release in the synapse and propagates an evoked potential to bipolar cells. Given the localization of the metabolic enzymes in monkeys, the same bipolar cells may be the main source of eCB production that will act in a retrograde manner and activate CB1R located in cone pedicles, thus regulating glutamate release. This eCB production will also synthesize 2-AG that will activate CB2R in Müller cells, thus modulating potassium spatial buffering throughout the retina. The activation of CB2R coupled to G_i/o_ will reduce the levels of cyclic AMP and PKA activity. Given that PKA activates KIR_4.1_ channels in Müller cells, CB2R will play a role by negatively modulating potassium. In scotopic conditions, the synaptic terminals of rods release a large quantity of glutamate. This glutamate binds to mGluR6 receptors located on the dendrites of ON rod bipolar cells. See Figure 6 for details.

### 2.2. Atypical Putative Cannabinoid Receptors GPR55 and TRPV1

To date, there are two atypical cannabinoid receptors that have been described in the monkey retina: (1) GPR55 has been found mainly in rods of the vertical pathway, and (2) TRPV1 in horizontal and amacrine cells of the horizontal pathway.

#### 2.2.1. Anatomical Localization and Function of GPR55

GPR55 is a GPCR that is naturally activated by LPI or LPG [42,43,44]. While the endogenous cannabinoid AEA and phytocannabinoid THC can also activate this receptor, the non-psychoactive compound CBD is an antagonist of this receptor [45]. GPR55 is an important modulator of retinal development in mice and hamsters [23]. GPR55 expression is widely expressed in the adult rodent retina [23], and it is exclusively expressed in rod photoreceptors, most prominently in inner segments of the vervet monkey retina [28] (Figure 4). Modulation of GPR55 by either blocking GPR55 (impaired retinal function, e.g., nyctalopia) or by activating it (increased retinal function, e.g., hyper-scotopia) impacts solely scotopic retinal function [41] (Figure 5).

#### 2.2.2. Anatomical Localization and Function of TRPV1

Transient receptor potential vanilloid type 1 (TRPV1) is a cannabinoid-like non-selective cation channel receptor that is the main target of the pungent compound found in hot chili peppers capsaicin [46]. In neurons, endovannilloids and endocannabinoids, like anandamide, 2-arachidonoylglycerol and N-arachidonoyl dopamine bind to TRPV1 [47]. Evidence for TRPV1 expression and function in the retina of multiple species has also been put forward [48]. In the goldfish and zebrafish retinas, TRPV1 was found in amacrine cells, as well as the synaptic ribbons of photoreceptors [49,50]. In the rat retina, TRPV1 was found in microglial cells, blood vessels, and astrocytes and in neuronal structures such as synaptic boutons of both plexiform layers as well as in cell bodies of the INL and GCL [51]. TRPV1 mRNA was detected in ganglion and Müller cells in the rat retina [26,52]. In the rabbit and human retina, TRPV1 is intensely expressed in the RPE [53]. TRPV1 is also present in the outer nuclear layer (ONL) and INL and at the end of the nerve fiber layer as well as in Müller cells of the rabbit retina [53]. In the vervet monkey retina, TRPV1 was found in the OPL and IPL, and in RGCL with a higher density in the periphery [27]. Co-immunolabeling of TRPV1 with parvalbumin, a primate horizontal cell marker, revealed a clear overlap of expression throughout the entire cell structure with most prominent staining in the cell body membrane and synaptic terminals. Furthermore, double labeling of TRPV1 and syntaxin was found throughout amacrine cells in the inner plexiform layer. Finally, double staining of TRPV1 and Brn3a allowed us to confirm its previously reported expression in the cell bodies and dendrites of RGCs [26]. The presence of TRPV1 in the horizontal pathway suggests a function of this receptor in lateral inhibition between photoreceptors through the horizontal cells, and between bipolar cells through amacrine cells (Figure 4). A role for TRPV1 channels in physiopathological retinal processes has been first ascribed in the rat retina [54]. Activation of TRPV1 has been then proposed to modify retinal protein expression patterns contributing to calcium-dependent signaling that maintains excitatory signaling in RGCs using TRPV1 knockout mice [26]. Inhibition of TRPV1 prevented retinal angiogenesis in a mouse model of oxygen-induced retinopathy [55,56]. We have not studied the role of TRPV1 in the retina of vervet monkeys. A good way to test this would be to perform intraocular injections of AM404 acting as a neuronal TRPV1 agonist, and SB-705498 acting as an antagonist in vervet monkeys, since the retina has no nociceptors [57].

#### 2.2.3. Interspecies GPR55/TRPV1 Comparison

Anatomical data on GPR55 retinal expression stems largely from two studies on mice and vervet monkeys [23,28]. To our knowledge, there has not been in other immunohistochemical study reported since the available and specific GPR55 markers are scarce. TRPV1 expression pattern is different in the retina of mice, macaque monkeys, and humans [26].

## 3. Cannabinoid Receptors in Retinal Diseases

Eye-related vision loss can occur because of damage of the optical system (e.g., corneal and lens anomalies due to multiple factors such as age, genetics, and intrusion of a foreign compound) or pathology of the neuro-retina (e.g., photoreceptors, bipolar cells, ganglion cells physiological dysfunction). While surgical interventions can treat pathologies of the optical system (cataracts, corneal opacifications…), there are little approved clinical treatments for retinal diseases like age-related macular degeneration (AMD), diabetic retinopathy (DR), glaucoma, and retinitis pigmentosa (RP). However, intense research is being carried out targeting these pathologies using a wide variety of approaches [58,59,60,61,62,63]. Current strategies focus on slowing down or stop the initial triggers (e.g., AREDS supplementation, exercise, eating well, and smoking cessation in dry AMD, and anti-VEGF intraocular injections in wet AMD) [64,65], or focus on relieving the symptoms (e.g., lowering intraocular pressure (IOP) with prostaglandin analogues or beta blockers in glaucoma) [66]. Given the wide expression profile of the eCB system in the neuro-retina, the use of this system as a pharmacological target, particularly the typical and atypical cannabinoid receptors, in the management of these retinal diseases is of great interest. Concomitantly, data obtained from preclinical studies shows that cannabis and cannabinoid molecules used as neuroprotective agents may have potential benefits to prevent glaucoma and other retinal neurodegenerative diseases [67]. The purpose of this review was not to exhaustively list the evidence supporting the idea that cannabinoids are useful in the treatment of retinal diseases, rather, to present how typical and atypical cannabinoid receptors can be used as novel pharmacological targets. Indeed, the potential of cannabinoids as anti-inflammatory, neuroprotective, or IOP lowering agents in the management of some retinal diseases has been reviewed elsewhere [68]. Given that cannabinoids can activate typical CB1R and CB2R, as well as atypical GPR55 and TRPV1, these studies can definitely serve as the basis of using for further drug development. A current clinical trial is studying if cannabinoids may be beneficial in certain degenerative diseases of the retina by determining whether cannabis derivatives affect the visual functions in healthy adults, and examining the effect of cannabis derivatives on the retina of retinitis pigmentosa patients using the ERG as the primary outcome measure, as well as other optometric tests (https://clinicaltrials.gov/ct2/show/NCT03078309, accessed on 12 November 2021).

### 3.1. Age-Related Macular Degeneration (AMD)

Macular degeneration is an eye disease typically due to normal aging. It is estimated that 9% of the worldwide population has AMD with a projected number of people with the disease around 196 million in 2020, and increasing to 288 million in 2040 [69]. More than 1 million Canadians are affected and is the leading cause of visual deficiency in the elderly. The macula is located in the center of the retina and allows for central vision and detection of fine details. AMD is thus the degeneration of the macula. Blurred central vision makes daily activities such as reading and driving difficult. Peripheral vision is often preserved as AMD affects primarily central vision. There are two forms of this disease, the dry and wet AMD. Dry AMD, the most frequent form, is caracterized by thinning or pigmentation/coloration of the macula. Wet AMD has a rapid and severe onset and is caused by abnormal forming blood vessels under the macula. The resulting flow of liquid/blood destroys the macular neuro-retina. THC acting on typical and atypical receptors has been shown to inhibit VEGF pathways, slowing down retinal angiogenesis in mice [70]. Cannabidiol by activating A_2A_ adenosine receptors in retinal microglial cells can as an anti-inflammatory agent in rats [71]. Finally, cannabidiol acting upon GPR55 could act as an inhibitor of cytokine production and inflammation, thus decreasing the thinning and degeneration of the macular region in monkeys [72].

### 3.2. Diabetic Retinopathy (DR)

Diabetes is a hormonal disease primarily affecting the control of sugar in the blood. High glucose blood levels reach the retinal circulatory system and can make tiny blood vessels swell and cause retinal detachment. This is called non-proliferative DR. New blood vessels can also start forming and compromise normal vision. This is known as proliferative DR. The resulting inflammation and oxidation, as well as degeneration of the nearby neuro-retina can worsen the prognostic of this disease. Anti-VEGF intraocular injections can slow down and stop newly forming blood vessels. Yet, there is no treatment targeting the resulting inflammation and neurodegeneration process occurring in DR. Modulating the eCB system (e.g., cannabidiol activating typical and atypical cannabinoid receptors) is a promising strategy for the treatment of DR by increasing anti-inflammatory processes and neuroprotection [73].

### 3.3. Glaucoma

Glaucoma is an eye disease that results in the destruction of the optic nerve neurons. Controlling the main risk factor that is IOP is an important strategy to slow down or prevent the disease. Cannabinoids consumed *orally* can lower IOP by activating cannabinoid receptors and probably the trabecular meshwork that controls the production and secretion of aqueous humor, but result in unwanted systemic side effects [74]. Specific typical and atypical cannabinoid receptor modulation in many delivery methods could be used as therapeutics to decrease inflammation and the resulting cell death [75].

### 3.4. Retinitis Pigmentosa

RP is an eye disease characterized by a slow degeneration of the pigmented epithelium in both eyes mainly caused by genetic factors. It is first manifested by night vision blindness and narrowing of the visual field. Central vision may also be affected tardively in some patients. Of importance is the role exerted by atypical GPR55 on improving scotopic vision. Given that RP affects night vision by altering rod photoreceptor function, exploiting agonists of GPR55 could have a tremendous impact on bettering good vision at an early stage of the disease, and possibly, slowing down the disease [28,41].

## 4. Prospects and Limitations

The progress in cannabinoid research might have an impact in the treatment of various retinal diseases. Indeed, recent studies have shown that the expression and localization of cannabinoid receptors in specific retinal components led to relate cannabinoid receptors to specific visual functions (photopic and scotopic vision). This prospect is highlighted by a study that clearly showed that an agonist of GPR55 (LPG) increased significantly the ERG response in scotopic conditions [41]. Given that night vison is impacted by various eye diseases, this compound offers a nice potetial therapeutic venue to restore normal night vision. Moreover, we are seeing now an increase in the discovery of new atypical cannabinoid receptors and ligands that will increase the number of potential therapeutic targets [76]. However, extending this research to humans proves to be difficult for methodological and ethical reasons. Nonetheless, given the strong similarity between the organisation of the retina and the visual system of primates and humans, data obtained from monkeys could be easily applied to humans.

## 5. Conclusions

**Figure 7:**
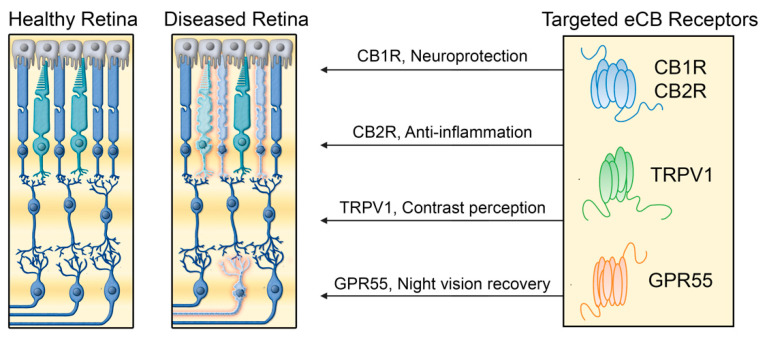
The main proposed mechanisms for the therapeutic effects of typical and atypical cannabinoid receptors in retinal diseases. Cannabinoid receptors can possibly contribute to treatment of retinal disorders by different mechanisms involving cannabinoid receptor type 1, cannabinoid receptor type 2, GPR55, and TRPV1 receptors.

We have clearly demonstrated that there is an anatomical segregation of the four different endocannabinoid receptors, CB1R, CB2R, GPR55, and TRPV1 in the retina of the vervet monkey. The development of new therapeutic agents that interact with typical and atypical cannabinoid receptors is crucial for potential treatment of retinal disorders (Figure 7). The four cannabinoid receptors CB1R, CB2R, GPR55, and TRPV1 are ideally positioned in the primate retina to play important functions. Modulation of CB1R in the neuro-retina can serve as neuroprotection. By activating CB1R in cones, bipolar cells, horizontal cells, amacrine cells, and ganglion cells, these retinal cells could be protected from AMPA excitotoxicity [77]. Modulation of CB2R in glial Müller cells can control many cytokine production, and serve as an anti-inflammatory component. By activating CB2R in Müller cells, the production of cytokines, nitric oxide, and/or reactive oxygen species might be controled [78,79]. GPR55 modulation can enhance the activity of rods, and increase night vision. By specifically activating GPR55 in rod photoreceptors, agonists of this receptor could act as photosensitizers to induce night vision enhancement, similarly to what was observed with porphyrins [80]. The activation could enhance TRPV1 modulation as a way to preserve contrast perception. By increasing lateral inhibition in horizontal cells, visual perception could improve by boosting the image contrasts [81].

## Data Availability

Data supporting reported results can be requested from J.B. (joseph.bouskila@umontreal.ca).
